# Supplementary material for: Identification of photoperiod-induced specific miRNAs in the adrenal glands of Sunite sheep (Ovis aries)
Source: Front Vet Sci. 2022 Jul 22;9:888207. doi: 10.3389/fvets.2022.888207 (PMC9354845; doi:10.3389/fvets.2022.888207)
Supplement: Supplementary file 1 [file Data_Sheet_1.ZIP › Supplementary file/miRDeep2 file.zip!/miRDeep2 file/Know_Structure/SPLP42A3/oar-mir-376e.pdf]

miRBase precursor	: oar-mir-376e
Total read count	: 673
oar-miR-376e-5p read count	: 173
oar-miR-376e-3p read count	: 327
oar-miR-376b-5p read count	: 173
remaining reads	: 0

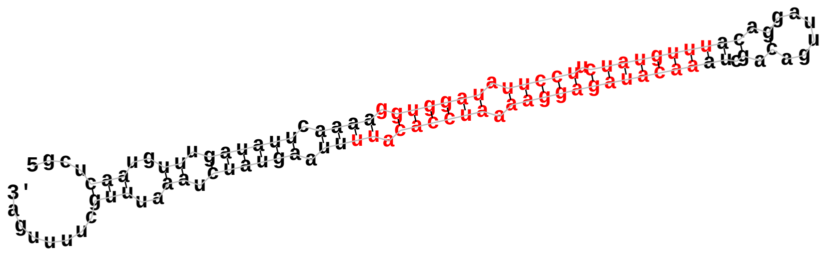

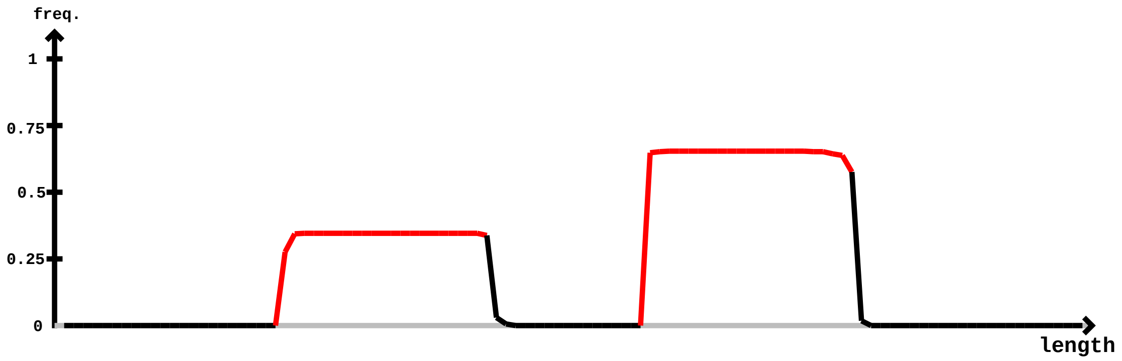


```
                                                                     oar-miR-376e-3p
                         oar-miR-376b-5p
                         oar-miR-376e-5p
5'- gcucaauguuugauauucaaaagguggauauuccuucuauguuuacaggauugacagcuaaacauagaggaaaauccacauuuuaaguaucuaaauuugcuuuuga  -3'    exp
    ((.....((((((((((.((((.((((((.(((((.((((((((((.(......).).)))))))))))))).)))))).)))).)))))).)))).))......    reads   mm    sample
    ......................gguggauauuccuucuauguu...............................................................    4       0     seq
    ......................gguggauauuccuucuauguuu..............................................................    125     0     seq
    ......................gguggauauuccuucuauguuua.............................................................    9       0     seq
    .......................guggauauuccuucuauguuu..............................................................    28      0     seq
    .......................guggauauuccuucuauguuua.............................................................    3       0     seq
    .......................guggauauuccuucuauguuuac............................................................    3       0     seq
    ........................uggauauuccuucuauguuu..............................................................    1       0     seq
    ............................................................aacauagaggaaaaucc.............................    1       0     seq
    ............................................................aacauagaggaaaauccac...........................    4       0     seq
    ............................................................aacauagaggaaaauccaca..........................    3       0     seq
    ............................................................aacauagaggaaaauccacau.........................    31      0     seq
    ............................................................aacauagaggaaaauccacauu........................    278     0     seq
    ............................................................aacauagaggaaaauccacauuu.......................    7       0     seq
    .............................................................acauagaggaaaauccacauu........................    1       0     seq
    .............................................................acauagaggaaaauccacauuu.......................    1       0     seq
    ..............................................................cauagaggaaaauccacauuu.......................    1       0     seq
```
